# Gonadal Hormones and Retinal Disorders: A Review

**DOI:** 10.3389/fendo.2018.00066

**Published:** 2018-03-02

**Authors:** Raffaele Nuzzi, Simona Scalabrin, Alice Becco, Giancarlo Panzica

**Affiliations:** ^1^Eye Clinic, Department of Surgical Sciences, University of Turin, Turin, Italy; ^2^Laboratory of Neuroendocrinology, Department of Neuroscience Rita Levi-Montalcini, University of Torino, Torino, Italy; ^3^Neuroscience Institute Cavalieri-Ottolenghi (NICO), Orbassano, Italy

**Keywords:** gonadal hormones, estrogens, hormone therapy, eye disorders, retinopathies, optic nerve, age-related macular degeneration, sex-related differences

## Abstract

**Aim:**

Gonadal hormones are essential for reproductive function, but can act on neural and other organ systems, and are probably the cause of the large majority of known sex differences in function and disease. The aim of this review is to provide evidence for this hypothesis in relation to eye disorders and to retinopathies in particular.

**Methods:**

Epidemiological studies and research articles were reviewed.

**Results:**

Analysis of the biological basis for a relationship between eye diseases and hormones showed that estrogen, androgen, and progesterone receptors are present throughout the eye and that these steroids are locally produced in ocular tissues. Sex hormones can have a neuroprotective action on the retina and modulate ocular blood flow. There are differences between the male and the female retina; moreover, sex hormones can influence the development (or not) of certain disorders. For example, exposure to endogenous estrogens, depending on age at menarche and menopause and number of pregnancies, and exposure to exogenous estrogens, as in hormone replacement therapy and use of oral contraceptives, appear to protect against age-related macular degeneration (both drusenoid and neurovascular types), whereas exogenous testosterone therapy is a risk factor for central serous chorioretinopathy. Macular hole is more common among women than men, particularly in postmenopausal women probably owing to the sudden drop in estrogen production in later middle age. Progestin therapy appears to ameliorate the course of retinitis pigmentosa. Diabetic retinopathy, a complication of diabetes, may be more common among men than women.

**Conclusion:**

We observed a correlation between many retinopathies and sex, probably as a result of the protective effect some gonadal hormones may exert against the development of certain disorders. This may have ramifications for the use of hormone therapy in the treatment of eye disease and of retinal disorders in particular.

## Introduction

There is a growing body of evidence for the importance of gonadal hormone action in the function of the reproductive and other systems (1), including bone (2) and cardiovascular system. Sex hormones (androgenic, estrogenic, and progestinic) are produced by both sexes, though the quantity and mode differ by sex and age. Moreover, they are produced, not only by the gonads, but also by other organs (3, 4), including the central nervous system (CNS) in which estrogens are thought to exert a neuroprotective role (5, 6).

Historically, interactions between gonadal hormones and the eye have received scarce attention; however, recent research into sex-related differences has begun to reveal possible links between estrogens and eye diseases, i.e., glaucoma, age-related macular degeneration (AMD), and cataracts. This has carried over into the evaluation of the implications that postmenopausal hormone replacement therapy (HRT) and anti-estrogenic therapy in breast cancer could have for concomitant eye disorders (7).

Since, research in this area is still at its beginning, the available studies are few and often limited in sample size; this does not allow to reach a univocal and definitive answer about the relationship between sex, sex hormones, and ocular pathologies. The purpose of this review is, therefore, to summarize the results currently present in the literature.

## Basics of Biology and Epidemiology of Interaction Between Gonadal Hormones and the Eye

### Presence of Hormone Receptors in the Human Eye

The eye was long considered a “sexually neutral” structure, meaning that it was believed that there were no differences in ocular physiology and pathology between the sexes. Today, however, we know that differences among sexes exist both in the physiology and in the pathology of the eye. In fact, the eye is a target for sex steroid hormones as demonstrated by the large presence of sex steroid hormone receptors (SSHRs). The SSHRs’ mRNAs are present everywhere in the eye (8): cornea, lens, iris, ciliary body, retina, lacrimal gland, meibomian gland, conjunctiva [for a complete list of citations see Ref. (9)]. In all these locations, estrogen receptor α (ERα), estrogen receptor β (ERβ), progesterone receptor, and androgen receptor (AR) mRNAs have been detected.

The distribution of SSHRs in the eye varies by sex and age, which partly explains the difference in the epidemiology of certain eye diseases (9). PCR assay, Western blot, and immunohistochemical analysis have demonstrated the presence of ER-α protein in the retina and RPE of young women, but not in postmenopausal women or men (10).

In addition to AR mRNA (8), the AR protein is present in the lachrymal and meibomian glands, the cornea, the bulbar conjunctiva, the lens, and the RPE, together with 5α-reductase (the enzyme converting testosterone into the more powerful dihydrotestosterone, DHT) type 1 and 2 mRNA (8).

### Synthesis of Steroids in the Retina

The mammalian retina has the ability of synthetize neurosteroids (pregnenolone, progesterone, dehydroepiandrosterone, desoxycorticosterone, 3 alpha, 5 alpha-tetrahydrodesoxycorticosterone, 3 alpha-hydroxy-5 alpha-dihydro-progesterone, 17-hydroxyprogesterone, and 17-hydroxypregnenolone) from cholesterol, as demonstrated by using retinal explants, thus excluding interferences from circulating steroids (11).

**Figure 1:**
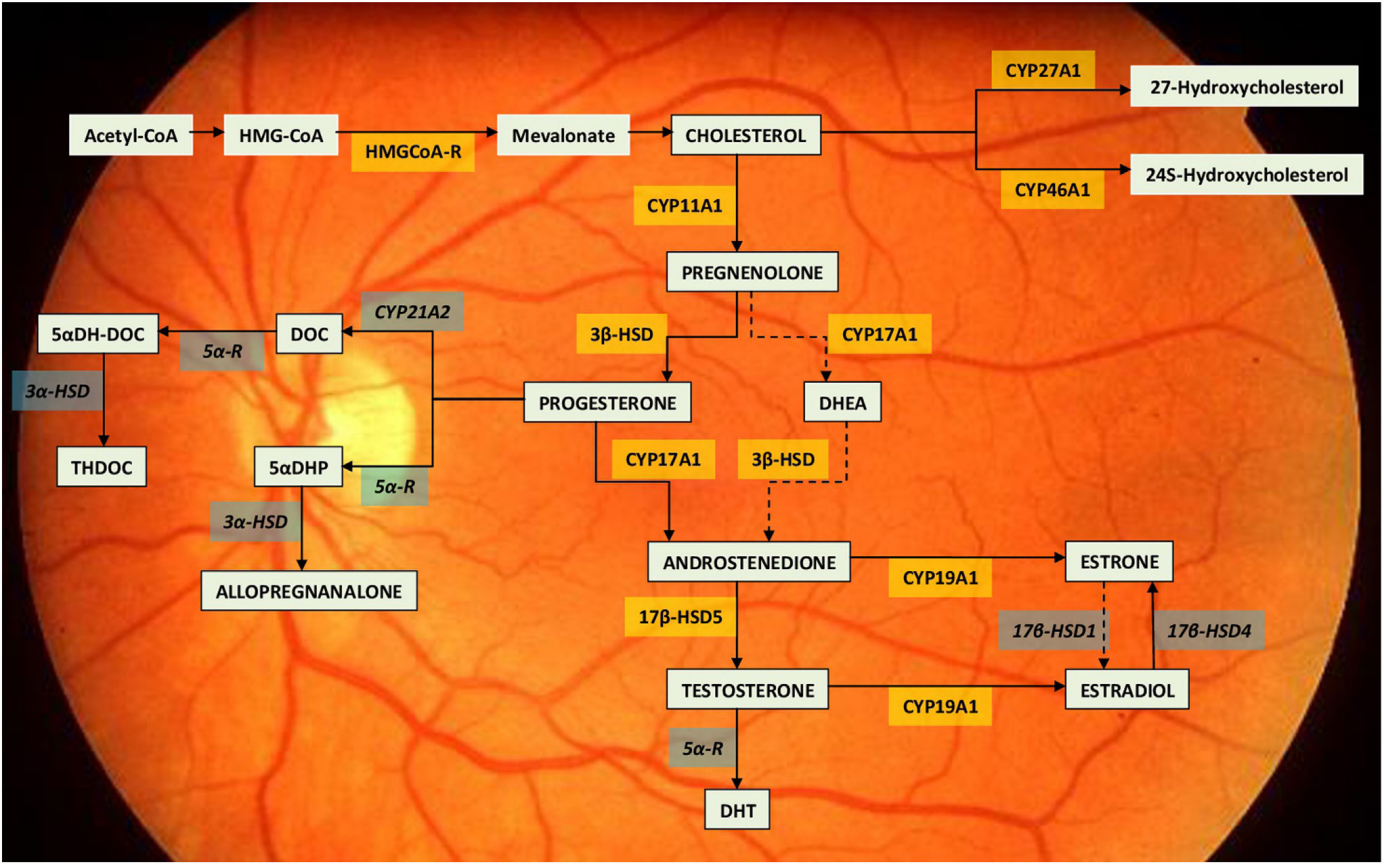
Neurosteroid synthesis in the retina. The drawing report of the metabolic pathway leading to estradiol synthesis within the retina (11, 14). The steroidogenic enzymes already identified for their mRNA, activity or immunolocalization are indicated within yellow boxes. The enzymes still lacking of identification are indicated within the grey boxes. Dotted lines indicate so far unclear enzymatic activities. Abbreviations: 3β-HSD, 3β-hydroxysteroid dehydrogenase; 3α-HSD, 3α-hydroxysteroid dehydrogenase; 5αDH-DOC, 5 alpha-dihydrodeoxycorticosterone; 5αDHP, 5α-dihydroprogesterone; 5α-R, 5α-Reductase; 17β-HSD1, 17β-hydroxysteroid dehydrogenases 1; 17β-HSD4, 17β-hydroxysteroid dehydrogenases 4; 17β-HSD5, 17β-hydroxysteroid dehydrogenases 5; DHEA, dehydroepiandrosterone; DHT, dihydrotestosterone; DOC, deoxycorticosterone; HMG-CoA, hydroxymethylglutaryl-CoA; HMGCoA-R, hydroxymethylglutaryl-CoA reductase.

Following studies demonstrated also the presence of steroidogenic enzymes (mRNA and protein) in the retina (12): cytochrome CYP11A1 (CYP450scc) that converts cholesterol in pregnenolone; 3-β-hydroxysteroid dehydrogenase which converts pregnenolone in progesterone; cytochrome CYP17A1 (P450c17) involved in the production of 17-α-hydroxymetabolites; and CYP19A1 (P450 aromatase) which converts testosterone in 17-β-estradiol [(11, 13, 14), Figure 1]. Cholesterol, which activates the metabolic cascade leading to the production of E_2_, is also produced in the retina: HMG-CoA reductase, the main enzyme for cholesterol synthesis, is present in the RPE, photoreceptors, and Müller cells. Exogenous cholesterol, on the other hand, is derived from high-density and low-density lipoproteins (HDL and LDL, respectively) that bind to specific receptors of the retinal cells (15). The principal limiting steps for estrogen production in the retina are the regulation of CYP450scc and aromatase enzymatic activities (14) (Figure 1). The steroidogenic enzymes are found in retinal neurons, glial cells, and photoreceptors in amounts similar to those observed in other part of the CNS (16). Enzymes’ concentration is greatest in the internal nuclear layer, which is considered the principal site of retinal steroids’ synthesis (14).

Finally, it is important to note that estrogens and androgens are also produced outside the retina (gonads, adrenals) and, through the blood flow, they can reach the eye where, in males, circulating testosterone may be locally metabolized in 17-β-estradiol. Diseases or functional alterations linked to steroid hormones may, therefore, be due to both retinal steroidogenic enzyme failure (short-term effects?), alterations in the gonadal and adrenal hormone supply (long-term effects?), or both conditions.

### Differences in Retinal Function between Men and Women

There are marked sex-related differences in ocular anatomy and pathophysiology, particularly for the retina. Studies on mice have identified differences in retinal structure between males and females and found that, as measured by multifocal electroretinography (mfERG), retinal function is better in females of reproductive age than in males and older females (17). Similar mfERG studies on humans found a statistically significant difference in neuroretinal function between men and women below 50 years, but not after this age; in addition, neuroretinal function was lowest in women who received a hysterectomy during reproductive age, with subsequent iatrogenic-induced menopause. These findings suggest that the estrogenic cycle has a beneficial effect on neuroretinal function and that estrogens may have a protective role (18).

### Sex-Related Differences in the Prevalence of Eye Disorders

Sex-related differences in eye anatomy and physiology are reflected in disease processes (19). Cataracts, for example, are far more prevalent among women than men: the prevalence of lens opacities in women aged between 65 and 74 years is 24–27%, but only 14–20% in their male counterparts (20–23). Estrogen levels, besides other risk factors, appear to play a role. Numerous studies have shown that HRT is a protective factor in postmenopausal women and that late menopause or early menarche, both of which augment estrogen exposure, lower the risk of cataracts in advanced age (24–27). The main protective action of estrogens on the lens is probably due to their antioxidant properties (28). With the onset of menopause, the drop in estrogen levels increases the risk of cataracts in older women. Differently, the levels of estrogen converted by aromatase from testosterone in men do not seem to be age dependent, affording men greater protection against the development of cataracts (29). This sex-related difference in eye disorders is not always so distinct. For example, sex is not considered a factor in AMD (30, 31), though some studies have suggested its higher prevalence among women (32, 33). When distinguished by type of AMD, neovascular (34) and drusenoid (32) forms are more prevalent among women. As reported for cataracts, longer exposure to estrogens or HRT confers a lower risk of developing AMD in later age (35, 36), suggesting that this is due to the antioxidant and anti-inflammatory actions of estrogen (37, 38).

Glaucoma is also more frequent in women (39), though this is more likely linked to the longer life expectancy of women and as such is an age-related risk factor (40). Nonetheless, sex-related differences have been associated with different types of glaucoma: a study conducted on an Asian population (39) reported that angle-closure glaucoma is more prevalent among women; other studies reported the same findings for white and black populations (41, 42), but other studies found that, after adjusting for age and population, angle-closure glaucoma is more common among men (40, 43). Sex-related differences in the prevalence of diabetic retinopathy are associated with the difference in the prevalence of diabetes. Though more men than women are affected by type 1 diabetes (44) no study to date has found a significant difference in its prevalence (45, 46). Similarly, no statistically significant sex-related differences in the prevalence of diabetic retinopathy associated with type 2 diabetes have been established, though some studies have suggested that it is more frequent among men (47, 48).

Various other risk factors besides sex alone need to be taken into account (49). For example, in a study on a rural south Indian population, Nirmalan et al. (50) tried to understand if female reproductive factors (age at menarche and menopause, number of pregnancies, etc.) were related with eye diseases. The study found no association with cataracts, open-angle glaucoma, macular degeneration, or myopia; nonetheless, the study presented several limitations, including the fact that the data on reproductive factors were gleaned from self-report questionnaires and that eye disorders were diagnosed in most subjects during the course of the study.

## Neuroprotective Effect of Sex Hormones

### Preclinical Studies

Several studies have investigated whether estrogens have a neuroprotective role and, if so, through which mechanisms they exert such action. Nixon et al. (51) examined the role of hormones in elevated levels of glutamate, which has a neurotoxic effect. High glutamate concentrations inhibit the cysteine/glutamate transporter, which reduces the production of glutathione, an antioxidant. This decrease leads to the augmented production of reactive oxygen species (52), which, owing to oxidative damage, promote the development of eye diseases, such as AMD (53) and retinitis pigmentosa (54). The experiment was carried out *in vitro* on 661 W cells, i.e., a mouse cone photoreceptor cell line, and demonstrated that E_2_ and the non-feminizing estrogen analogs ZYC-26 and ZYC-3 exert a protective action against the damage induced by 5 µM of glutamate. It was also observed that the protective effect of ZYC-26 and ZYC-3 is not exerted *via* the classic estrogen receptors ERα and ERβ, as demonstrated by the persistent protective action despite the use of an estrogen receptor pan-antagonist (ICI182780) and the lack of a protective effect after the use of the two agonists of ERα and ERβ. Based on these results, it was hypothesized that non-feminizing hormones could be used in the treatment of neurodegenerative eye disorders, and thus avert the side effects of prolonged estrogen therapy. Mo et al. (55) investigated intracellular neuroprotective mechanisms in ovariectomized mice, as measured by electroretinography of light-induced apoptosis in retinal cells. Following intravitreal administration of 17β-estradiol, retinal function was preserved due to the reduction of neuronal apoptosis. The involved pathway is PI3k/Akt activation: administration of a PI3K inhibitor (LY294002) increases retinal neuronal apoptosis, while the administration of estrogens leads to the translocation of NF-kB p65 from the cytosol to the nucleus, which is inhibited in the presence of LY294002. These results demonstrated that the protective action of estrogen on the retina is exerted *via* activation of the PI3K/Akt cascade and concludes with the nuclear translocation of NF-kB (55). The mechanism of action reported in this study is not the only one through which sex hormones exert their neuroprotective effect.

Estrogens also have a protective effect on intraretinal synapses. Kaja et al. (56) used a mouse model in which mild retinal ischemia was induced by transient occlusion of the middle cerebral artery. This experimental condition is ideal for examining the earliest stages of retinal damage that precede the development of neurodegenerative processes. Synaptic activity was measured using an immunoreactive technique based on the detection of Vesl-1L7Homer 1c (V-1L), a neuronal cytosolic protein involved in receptor clustering for neurotransmitters and in neuronal development and plasticity. V-1L is also a good marker to evaluate the changes in synaptic connectivity during the early stages of apoptosis of retinal ganglion cells. The study (56) found that retinal ischemia, though mild, can significantly reduce the number of V-1L-positive synapses in the internal plexiform layer of the retina, and increase the number of neuronal apoptotic cells in the ganglion cell layer. Estrogen administration exerts a protective effect by reducing the percentage of cells undergoing apoptosis and by preventing early ischemia-induced changes preceding apoptosis in the synaptic connections.

The neuroprotective action of estrogens appears to be closely linked to their antioxidant activity. Among the studies investigating this property, the early study by Moosmann et al. (57) merits mention as it demonstrated that the antioxidant-neuroprotective action of the hormones is not due to their genomic property, i.e., their ability to influence the transcription of specific genes, but rather because of their chemical properties as hydrophobic phenolic molecules. Estrogens were compared with other phenol molecules: the results showed that the protective effect against glutamate-induced oxidative toxicity in neuronal mouse cells was present in all the compounds studied and that the dose to obtain this effect was also the same. The study also showed that there is no correlation between estrogen strength and its antioxidant properties. The discovery of the dissociation between the hormonal effect and the neuroprotective effect could open the way to the development of new therapies using molecules that possess the same protective properties as estrogens, but without the unwanted effects associated with their activities as sex hormones.

Nakazawa et al. (58) investigated differences in neuroprotective activity between “endogenous” estrogens (produced in ovary) and “exogenous” estrogens (administrated through intravitreal injection) in a mouse model by comparing their neuroprotective effects against retinal ganglion cell (RGC) death following axotomy of the optic nerve, which mimics glaucoma-induced RGC death. To evaluate endogenous estrogen activity, samples of retinal tissue from female mice, ovariectomized or not, served as controls in which RGC density was measured. While ovariectomy had no effect on RGC density, the density was significantly reduced in mice that received ovariectomy and axotomy as compared to those that received only axotomy. These findings underscored the neuroprotective role of endogenous estrogens. In the evaluation of exogenous estrogens, ovariectomized mice administered intravitreal 17β-estradiol had a reduction in RGC death correlated with axotomy. The neuroprotective role of estrogens in this experimental condition indicates the potential for estrogen therapy in persons with etiologically similar eye diseases, such as glaucoma. In the second part of the study, the researchers wanted to identify the mechanism by which the exogenous estrogens exert their neuroprotective action. Immunoblot assay and immunohistochemical analysis showed that following intravitreal administration of E_2_, activation of the ERK signal transduction pathway, and c-Fos was augmented, whereas no change in PI3K/Act activation was observed. This finding was then confirmed in an experiment using U1026, an ERK inhibitor. Administration of U0126 before administration of E_2_ inhibited the neuroprotective effect of the estrogens (58).

Another molecular mechanism by which estrogens exert their neuroprotective effect involves upregulation of stromal cell-derived factor 1 (SDF-1). SDF-1 protects against retinal ischemia *via* its powerful chemotactic effect that promote tissue repair and the migration of stem cells produced in bone marrow to the site of damage (59). To demonstrate this factor’s mechanism of action, transient retinal ischemia was induced by increasing intraocular pressure (IOP) to 110 mm Hg in a mouse model. Following reperfusion, the expected increase in mRNA and SDF-1 was measured *via* real-time PCR and Western blot. To evaluate the role of estrogens, E_2_ was administered peripherally before inducing ischemia and SDF-1 was measured. The increased quantity of SDF-1 indicated that also in this condition estrogens play a neuroprotective role in reducing retinal damage (60).

Several studies aimed to determine whether only estrogens exerted neuroprotective action or other sex hormones, such as progesterone possessed similar properties (61). Progesterone was administered by peripheral infusion to one half of a population of male rats that had undergone photostress-induced retinal degeneration. Electroretinography showed no statistically significant differences between the two cohorts. The study findings suggested that progesterone has no protective effects similar to those of estrogens.

### Clinical Studies

In addition to preclinical animal studies, also clinical studies have been performed, including a population-based study in postmenopausal Korean women (62) in which the women were administered a questionnaire investigating their gynecological characteristics and whether they were taking estrogens as HRT. The women also received an eye examination, which revealed a higher prevalence of eye diseases, including anterior polar cataracts and other retinal disorders in the women not receiving HRT, suggesting its neuroprotective action.

## Effect of Sex Hormones on Ocular Blood Flow

Besides acting directly on retinal neuronal cells, sex hormones can also influence tissue perfusion by modulating retinal and choroid blood flow. A recent review of the literature (63) on sex differences in ocular blood flow sought to determine the possible role of sex hormones. The rationale for the study was derived from the fact that the eye diseases in which reduced blood flow is considered a causal or contributing factor, including AMD (64–66), glaucoma (67, 68), and diabetic retinopathy (69–71) are also those for which a sex-related correlation with prevalence has been found, suggesting that sex hormones may be implicated in the development of these diseases. Estrogens appear to play a protective role probably because of their vasodilatory action in reducing vascular resistance. This finding is shared by several studies that compared blood flow velocity and resistance of the ophthalmic artery and the central retinal artery in pre- and postmenopausal women. Blood flow velocity was higher and vascular resistance indices were lower in the premenopausal women (72), whereas vascular resistance of the central retinal artery was reduced after estrogen administration as compared to placebo (73). Retinal blood flow was higher in women receiving HRT than in those naïve to HRT (74); however, the evidence was not sufficiently strong to recommend HRT in the treatment of these conditions.

While estrogens exert a vasodilatory effect on retinal perfusion, testosterone, like progesterone, has the opposite effect (72). Progesterone exerts a vasoconstrictive effect on ocular blood flow. As demonstrated by color Doppler imaging (75), progesterone increases the resistance of ophthalmic and retinal arteries. In women of reproductive age, progesterone was found to antagonize the vasodilatory effect of estrogen during the menstrual cycle, as measured with the pulsatility index of the central retinal artery (76).

The role of HRT has been extensively studied because of the implications it can have for postmenopausal women with eye diseases. Postmenopausal women receiving HRT or not were compared with regards to blood flow in the inferotemporal retinal artery (ITRA), the peripapillary retina, and the margin of the optic nerve head, as measured using stereometric parameters and electroretinography (73). Blood flow in the ITRA was significantly higher and trophism of the optic nerve head and surrounding area was better in those receiving HRT. The effect of estradiol on retinal perfusion was investigated using ovariectomized mice; in this model E_2_ treatment improved retinal perfusion mainly through the increase in blood flow. Both HRT and administration of E_2_ exert a protective effect on the retina and the retinal nerve fiber layer by modulating tissue perfusion.

Harris-Yitzhak et al. (77) compared blood flow velocity in the retrobulbar arteries of postmenopausal women receiving HRT or not and young women of reproductive age. Hemodynamic resistance in the ophthalmic artery was lower in the young women and those receiving HRT than in the postmenopausal women not receiving HRT, whereas central retinal artery blood flow was similar for all three groups. Blood flow in the posterior ciliary arteries was better in the young women than in the two groups of postmenopausal women in which blood flow was similar. These findings suggested that HRT may modulate resistance in the ophthalmic artery, whereas its effect on other arteries is less pronounced, since changes in blood perfusion in these areas seem to be related to age.

Other studies have compared the effect of estradiol and testosterone on ocular hemodynamics by measuring the serum levels of the two hormones in pre- and postmenopausal women not receiving HRT, in addition to evaluating *via* color Doppler blood flow velocity and vascular resistance in the ophthalmic and central retinal arteries (72). The findings showed that peak systolic blood flow velocity in the ophthalmic artery correlated with serum estradiol levels, whereas vascular resistance of the central retinal artery decreased with increasing levels of estrogens in both groups of women. Peak systolic blood flow velocity correlated negatively with serum testosterone levels in the premenopausal women, whereas vascular resistance increased with higher testosterone levels. The two hormones were found to exert opposite effects: testosterone seemed to exert an antagonist effect as compared to estrogen.

The action of testosterone on ocular hemodynamics has also been studied in men, in which testosterone levels are naturally higher than in women. Low testosterone levels correlated with hypertension and higher cardiovascular risk (78, 79). There are also population-based differences. In fact, Malan et al. (80) described differences for the cardiometabolic prognosis and intraocular perfusion pressure in two cohorts of black and white men aged between 28 and 68 years. Only in white men there was a positive correlation between free testosterone levels and retinal vessel diameter (except for the central retinal artery in which the vessel diameter was inversely proportional to testosterone levels). The findings suggested a population-based protective effect of testosterone on vascularization and retinal perfusion, probably linked to the vasodilatory effect in the microvasculature.

## Diseases of the Retina, Optic Nerve, and Possible Sex-Related Effects

In this section of the review we tried, for each pathology, to collect the evidence in favor of the correlation with sex hormones and those against (Table 1).

**Table 1:** Role of sex hormones in ocular diseases.

++	+	+/−	/	−
Protective effect	Modest correlation	Non-significant correlation	Non-associated factor	Inversely correlated factor
Disease	E	P	T	Studies
AMD	+/*−*	/	/	Menopausal and reproductive factors and risk of age-related macular degeneration. Feskanich et al.
				The effect of the hormone therapy on the risk for age-related maculopathy in postmenopausal women. Abramov et al.
				Reproductive exposures, incident age-related cataracts, and age-related maculopathy in women: the Beaver Dam Eye Study. Klein et al.
				Female reproductive factors and eye disease in a rural south Indian population: the Aravind comprehensive eye survey. Nirmalan et al.
	/			Clinical risk factors for age-related macular degeneration: a systematic review and meta-analysis. Chakravarthy et al.
				Age-related macular degeneration guidelines for management. The Royal College of Ophthalmologists.
				Sex steroid and AMD in older French women: the POLA study. Defay et al.
AMD(drusenoid or neovascular)	++			Hormone therapy and age-related macular degeneration. The women’s health initiative sight exam study. Haan et al.
				Inverse association of female hormone replacement therapy (HRT) with AMD and interaction with ARMS2 polymorphisms. Velez et al.
				HRT, reproductive factors, and age-related macular degeneration: the Salisbury Eye Evaluation Project. Freeman et al.
				Association between reproductive and hormonal factors and age-related maculopathy in postmenopausal women. Snow et al.
	+			Clinical risk factors for age-related macular degeneration: a systematic review and meta-analysis. Chakravarthy et al.
				Risk factors for age-related macular degeneration. Evans
				Five-year incidence of age-related maculopathy lesions: the Blue Mountains Eye Study. Mitchell et al.
				Risk factors associated with age-related macular degeneration: a case-control study in the age-related eye disease study (AREDS): AREDS report number 3. Group, AREDS research
				Smoking, alcohol intake, estrogen use, and AMD in Latinos: the Los Angeles Latino Eye Study. Fraser-Bell et al.

CSCR	/	/	−	The potential role of testosterone in central serous chorioretinopathy. Grieshaber et al.
				Central serous chorioretinis associated with testosterone therapy. Ahad et al.
				Central serous chorioretinopathy in patients receiving exogenous testosterone therapy. Nudleman et al.
				Finasteride for chronic central serous chorioretinopathy. Forooghian et al.
	/	/	/	Serum cortisol and testosterone levels in chronic central serous chorioretinopathy. Tufan et al.
				Serous central chorioretinopathy and endogenous hypercortisolemia. Kapetanios et al.

MACULAR HOLE	++	/	/	Clinical features of idiopathic macular cysts and holes. McDonnell et al.
				Macular holes. James and Feman
				Estrogen antagonist and development of macular hole. Chung et al.
				Estrogen and macular holes: a postal questionnaire. Gray et al.
				Systemic risk factors for idiopathic macular holes: a case-control study. Evans et al.
	*−*	/	/	Vitreous estrogen levels in patients with an idiopathic macular hole. Inokuchi et al.

Retinitis Pigmentosa	/	++	/	Enhancing survival of photoreceptor cells *in vivo* using the sintetic progestin norgestrel. Doonan et al.
				Norgestrel may be a potential therapy for retinal degenerations. Doonan and Cotter
				Neuroprotective actions of progesterone in an *in vivo* model of retinitis pigmentosa. Sanchez-Vallejo et al.

Diabetic Retinopathy	/	/	+/−	The role of sex hormones in diabetic retinopathy. Grisby et al.
				Dehydroepiandrosterone protects bovine retinal capillary pericytes against glucose toxicity. Briganrdello et al.
	++ *If early stage* − *If late stage*		/	Gender and estrogen supplementation increases severity of experimental choroidal neovascularization. Espinosa-Heidmann et al.
				Effects of tamoxifen versus raloxifene on retinal capillary endothelial cell proliferation. Grigsby et al.
	/	/	/	Exogenous estrogen exposures and changes in diabetic retinopathy: the Wisconsin Epidemiologic Study of Diabetic Retinopathy. Klein et al.

Glaucoma	++	/	/	Is estrogen a therapeutic target for glaucoma? Dewundara et al.
				The obligatory role of endothelial cells in the relaxation of arterial smooth muscle by acetylcholine. Furchgott et al.
				Estrogen deficiency accelerates aging of the optic nerve. Vajaranant and Pasquae
				Primary open-angle glaucoma. Weinreb and Khaw
				The effect of the menstrual cycle on optic nerve had analysis in healthy women. Akar et al.
				Estrogen pathway polymorphisms in relation to primary open angle glaucoma: an analysis accounting for gender from the United States. Pasquale et al.

*For and against evidences for each disease, with type of correlation*.

*AMD, age-related macular degeneration; CSRC, central serous chorioretinopathy; E, estrogens; P, progesterone; T, testosterone*.

### Age-Related Macular Degeneration

#### Evidence for the Protective Effect of Estrogens

Age-related macular degeneration is a progressive multifactorial eye disease that leads to deterioration of vision, loss of spatial and color vision, and adaptation to darkness (81–85). According to the World Health Organization, AMD is the cause of blindness in 10% of cases. Histological hallmarks are degeneration of the RPE, Bruch’s membrane, and the choriocapillaris, resulting in photoreceptor damage and death (86).

The pathogenesis of AMD is multifactorial: lipofuscin accumulation in the lysosomes of the RPE; extracellular drusen deposits between the RPE and the internal collagen layer of Bruch’s membrane; oxidative damage; and chronic inflammation. Owing to their antioxidant and anti-inflammatory properties, estrogens might play a protective role in AMD (38). Beside these pathogenic factors there are genetic and environmental risk factors: age is perhaps the most important, in addition to smoking, obesity, atherosclerosis, hypertensions, hypercholesterolemia, unhealthy diet, and history of cataract surgery (81). Female sex is a weak risk factor (32, 87, 88) though exudative AMD is more common among women (34). These risk factors have been investigated in various studies. A case-control study (33) involved the participants in the Age-Related Eye Disease Study (AREDS) and used as controls subjects with fewer than 15 small drusen. Preliminary analysis showed that age was the main risk factor; subsequent analyses were accordingly adjusted for age to minimize its confounding with other factors. Smoking and hypertension both resulted as risk factors. Other correlated characteristics were Caucasian race, body-mass index, low educational level, hypermetropia, lens opacities, and female sex. A population-based study (89) identified alcohol abuse as another risk factor. Exposure to exogenous estrogens was a weakly protective factor against drusenoid deposit in AMD.

Spurred by the prospects of a protective action by estrogens against the development of AMD, researchers investigated the effect of HRT in postmenopausal women. Haan et al. (90) sought to determine whether HRT had a beneficial effect and whether different HRTs achieved different effects. They compared the efficacy of therapy based on conjugated equine estrogens (CEE) with CEE therapy combined with a progestinic. No association was found between the use of either therapy and the early development of AMD, suggesting that the early stages of AMD are not influenced by HRT. In contrast, conjugate therapy was more effective than CEE therapy alone in reducing the risk of developing both the drusenoid and neovascular forms of AMD.

In addition to investigating the protective effect of HRT on the development of certain types of AMD, Feskanich et al. (91) examined the potential role of estrogens as oral contraceptives during reproductive age. They observed a lower risk for the development of neovascular AMD in women receiving HRT. The risk was further reduced in those who, in addition to HRT, had also taken oral contraceptives. An unexpected result was the correlation between the risk of early AMD and HRT. The risk of early AMD was higher in the women who had received HRT; this contrasted with other studies that reported no correlation between the two factors. When the gynecological characteristics (age at menarche and menopause, number of pregnancies) were analyzed, none of these reproductive factors significantly modified the risk of developing AMD, except for a slight reduction associated with multiple pregnancies.

Velez Edwards et al. (92) investigated whether genetic factors could interact with HRT in modulating the risk of AMD and found that postmenopausal HRT and use of estrogen oral contraceptives during reproductive age had a protective effect against AMD. When, however, the study population was stratified by AMD severity, i.e., distinguishing between early AMD characterized by geographic atrophy and neovascular AMD, the lower risk remained only for neovascular AMD. During the second part of the study, genetic analyses were carried out on peripheral blood samples to determine whether single nucleotide polymorphisms (SNPs) in genes thought to increase the risk of AMD could modulate the risk of AMD in association with HRT or previous exposure to oral contraceptives. The study findings showed that two SNPs of the *ARMS2* gene (AMD 2) located on chromosome 10 enhanced the positive effect of HRT in preventing AMD.

The hypothesis for a link between HRT and lower AMD incidence was investigated by other studies which showed that estrogen exerted a protective effect only against certain types of AMD, like drusenoid AMD, demonstrating that this type of AMD is more prevalent among women with multiple pregnancies, whereas the correlation was not statistically significant when comparing early AMD and late AMD (93).

Other studies focusing attention on the role reproductive factors can have in the incidence of maculopathies. Blasiak et al. (37) reported a statistically significant reduction in the incidence of advanced AMD in women who had received HRT and a significantly higher risk of developing advanced AMD in women who began menarche late. These findings demonstrated that estrogen exposure, including exogenous estrogens, plays a beneficial role in reducing the risk of advanced AMD. The hypothesis that early menopause (before age 45 years), because it reduces the duration of exposure to estrogens, could constitute a risk for the development of macular degeneration was evaluated by Vigerling et al. (94) who compared different samples of women, including those that had experienced spontaneous early menopause and others who had experienced iatrogenic early menopause following ovariectomy. The results showed no increased risk for AMD among the women who experienced spontaneous early menopause, whereas the risk of developing macular degeneration was significantly increased in those who had undergone ovariectomy before age 45 years.

#### Studies Showing No Protective Effect of Estrogens

While some studies have found a neuroprotective effect of estrogens, others have not, leaving the question of their potential neuroprotective effect open. The guidelines for the management of AMD issued by the Royal College of Ophthalmologists (95) state that, on the basis of a meta-analysis (32), female sex is not a factor associated with higher risk of AMD. The guidelines go on to state that the higher prevalence of AMD among women is largely due to their longer life expectancy (87).

Numerous studies cited by the guidelines report that reproductive characteristics, e.g., age at menarche and menopause, and exposure to HRT are inconsistently associated with AMD (50, 96–98). The POLA study, for example, found that advanced AMD and drusenoid AMD or AMD with pigment abnormalities were not associated with oophorectomy or hysterectomy or HRT exposure. The POLA study examined serum levels of diverse hormones and correlated molecules (estradiol, testosterone, DHEAS, sex hormone-binding globulin) and found a correlation, albeit weak, only for high DHEAS levels that appeared to be associated with an increased prevalence of drusenoid AMD. Other studies and a review corroborate the hypothesis for the lack of a correlation between estrogens and macular degeneration. A systematic review (32) of 18 population-based studies and 6 case-control studies on the strength of the relationship among factors, increasing the risk for AMD reported that the risk factors most strongly correlated with degenerative maculopathy were age, smoking (33, 99, 100), history of cataract surgery (101–103), and family history of AMD (104, 105). Moderately correlated factors were body-mass index (99), history of cardiovascular disease (99, 106), hypertension (99, 106, 107), and elevated plasma fibrinogen (108). Factors weakly or inconsistently correlated with AMD were sex (107, 109, 110), ethnic group (107, 111), color of iris (104), history of cardiovascular disease (99, 106) and serum levels of cholesterol (99, 106), and triglycerides (106). Female sex was not considered a risk factor, precluding the role of sex hormones in higher risk for the disease.

### Central Serous Chorioretinopathy (CSCR)

#### Evidence of the Association between CSCR and Sex Hormones: Testosterone and Increased Risk of CSCR

Central serous chorioretinopathy is an acquired eye disease characterized by exudative detachment of the retinal and/or the RPE. Its pathogenesis is not yet fully understood; however, it is thought that alterations in choroid circulation and RPE function may be implicated in the development of the disease. This notion is corroborated by findings from fluorangiography and OCT that document augmented capillary permeability and pressure of the choroid vessels (107, 108). Risk factors vary widely from psychosocial stress to type A personality, Cushing’s syndrome, infections, smoking, alcohol, to pregnancy, and steroid therapy (109–111), all of which are characterized by elevated serum levels of glucocorticoids (112–114).

Given its higher prevalence among men, androgens have been directly implicated in the pathogenesis of CSCR (111, 115). In their study, Nudleman et al. (116) examined patients receiving exogenous testosterone therapy and who had no known risk factors for CSCR. The results showed that testosterone therapy is a probable independent risk factor for CSCR; moreover, disease symptoms and subretinal fluid accumulation resolved after discontinuation of testosterone therapy. Further evidence for the role of testosterone came from a case report of a woman who was not pregnant while receiving testosterone therapy (111), and another case report of a male population receiving testosterone therapy for hypogonadotropic hypogonadism (115), the latter of which demonstrated a temporal correlation between administration of therapy and the development of CSCR, indicating both testosterone and estrogen as the cause. Given the link between testosterone and CSCR, finasteride, an inhibitor of DHT synthesis, was considered as an alternative in the treatment of CSCR. Forooghian et al. (117) investigated the effect of finasteride administered for 6 months and monitored changes in visual acuity, macular thickness, subretinal fluid accumulation, OCT, serum DHT and testosterone, and cortisol levels in urine. Though no changes in visual acuity occurred, macular thickness and subretinal fluid level were lowest at 3 months in therapy before increasing slightly though remaining below basal reference limits. Furthermore, macular thickness and subretinal fluid both increased after therapy was discontinued in four patients and normalized in the patients who continued with therapy. These findings suggest a possible role for finasteride in the treatment of chronic CSCR.

#### Studies Showing an Absence of Association between CSCR and Testosterone

The study of Kapetanios et al. (114) reported a statistically significant association between elevated cortisol levels and the development of ICSCR, whereas no significant differences in testosterone levels were noted and levels remained within the normal range in both groups. A case-control study (118) of chronic ICSCR reported that cortisol and testosterone levels were similar in both groups. Though there appears to be a correlation between hormones and ICSCR, conflicting evidence leaves many questions about the etiopathogenesis and treatment of CSCR open.

### Macular Hole

#### Evidence of Estrogen Protection in the Macular Hole

Macular hole is a major cause of diminished vision, especially in advanced age. Although in some cases the cause can be identified, e.g., contusive trauma (119), cystoid macular edema (120), or diabetes (121), it is idiopathic in the majority and thought to be due to circumferential vitreoretinal contraction (122). Macular hole affects women far more often than men (123). As in other eye diseases, it is thought that estrogens have a beneficial effect on macular health and protect against the development of macular hole. For example, estrogens stimulate the synthesis of collagen and hyaluronic acid in the skin, suggesting that a similar process might also occur in the eye. With the sudden drop in estrogen production after menopause, this protection is lost, posing the retina to a higher risk than that for men, in whom estrogen levels are generally low throughout life and do not change abruptly. In addition to menopause, early hysterectomy or HRT may influence estrogen activity in relation to the development of macular holes (124, 125). Current evidence indicates strong correlations between macular hole and female sex and postmenopausal age (126), as demonstrated by various studies. A population-based study showed a female-to-male ratio of 3.3:1 for full-thickness macular hole (127). In their case-control study, Evans et al. (128) found that 67% of persons with macular hole were women and that 74% of the women were aged 65 years or older. The study analyzed various risk factors, including ethnic origin, systemic comorbidities, current use and history of medications, alcohol intake, smoking, body weight and height, menstrual and obstetric history, age at menopause and severity of associated symptoms, and exposure to HRT. The study findings indicated few systemic factors associated with idiopathic full-thickness macular hole (IFTMH), and though sex-correlated, no association was found between the principal indicators of exposure to estrogens and the incidence of macular hole. Nonetheless, a role for estrogens was suggested by the fact that women with macular hole generally experienced a more difficult menopause and more bothersome climacteric symptoms such as hot flashes than healthy women. The results also suggested that the development of IFTMH may be more due to the sudden change in hormone levels that chronic exposure, as demonstrated by the higher risk is associated with menopause, hysterectomy, and oophorectomy.

Correlations have been found between tamoxifen and macular hole and its precursor lesions (129). Tamoxifen is an anti-estrogenic nonsteroid drug used in adjuvant therapy for breast cancer (130). In this case report, all three women were receiving tamoxifen therapy and presented with cystic changes of the foveal region and defects of the external retina suggestive of initial macular hole.

#### A Study Shows That Estrogen Could Have a Negative Effect on Macular Hole

Another study investigated whether there were differences between the estrogen levels in the vitreous of subjects with macular hole and in those with other retinal disorders, who served as controls (131). The estrogen concentration was significantly higher in those with macular hole (*p* < 0.05), implicating it in the pathogenesis of the disorder. Since estrogens activate collagenase, this could be correlated with the development of vitreous collagen disorders; however, owing to the small study sample (10 cases and 9 controls), no definitive conclusions could be drawn.

### Retinitis Pigmentosa

#### Evidence of the Benefical Effect of Progestinic Therapy in Retinitis Pigmentosa

Retinitis pigmentosa refers to a group of inherited retinal degenerative disorders in which genetic mutations lead to the death of retinal photoreceptors (132). Although the mutations in several genes implicated in the development of the diseases are known, the mechanism by which they cause cell death is not fully understood and no effective treatment is currently available (133). Recent studies using mouse models have suggested Norgestrel, a synthetic analog of progesterone, as a potential agent (134). Norgestrel’s protective action has been demonstrated on retinal explants *in vitro* and in two different *in vivo* models of retinal degeneration: the one involving mice exposed to photostress-induced damage and the other involving mice with genetic mutations characteristic of retinitis pigmentosa. Both models showed a reduction in cell loss, with improvement in cell survival of about 70% (calculated on the basis of photoreceptor number, structural integrity, and function), indicating that apoptosis was interrupted or slowed. Although the mechanism of action is not yet fully understood, it is thought that norgestrel activates a survival pathway probably based on the increased expression of fibroblast growth factor 2 (FGF-2), a powerful neurotrophic factor whose production is increased by retinal stress. Its action promotes cell survival and inhibits apoptosis by activating the intracellular cascades involving mitogen-activated protein kinase, PI3K, and protein kinase C (PKC). The involvement of FGF-2 was demonstrated by measuring its changes in the control subjects who were administered only the vehicle and in the subjects receiving norgestrel therapy: the finding of higher FDG-2 values after norgestrel administration confirmed the hypothesis. These findings were strengthened by the same research group in a subsequent study (135) in which they investigated the potential of norgestrel as therapy for retinal degeneration.

Other studies investigated the relationship between progesterone and progestrinics and retinitis pigmentosa (136) building on previous studies on progesterone in experimental models of acute brain damage in which the drug had shown a neuroprotective effect (137). The action of progesterone was analyzed to determine the number of surviving cells, as measured by electroretinography, and the potential protective effect of the drug owing to its ability to limit damage by free radicals or to increase antioxidant defenses. The results showed a reduction in cell death and gliosis, with a statistically significant reduction in glutamate and a significant increase in reduced glutathione and oxidized glutathione. The study underlined the beneficial action of progesterone which exerts *via* multiple modes of action in protecting the retina during retinitis pigmentosa, suggesting its use or that of its analogs in the treatment of the disease.

### Diabetic Retinopathy

#### The Uncertain Role of Sex Hormones in Diabetic Retinopathy

Poor glycemic control ultimately results in macrovascular and microvascular complications, affecting the kidney and the eye in persons with diabetes. Diabetic retinopathy is most common complication (138) and is present in 34.6% of diabetics (139). Two forms are distinguished: nonproliferative or early stage diabetic retinopathy, and proliferative or late stage retinopathy in which retinal neovascularization is evident, leading to increased risk of loss of vision due to retinal detachment, neovascular glaucoma, and vitreous hemorrhaging. Another common cause of loss of vision is macular edema, which may arise in any stage of retinopathy (140). Studies investigating the possible links between sex hormones and diabetic retinopathy have analyzed the incidence of the condition by sex and produced conflicting results (49). One possible explanation for the discrepancies is the likely presence of confounding factors. Grisby et al. (141) compared the direct action of sex hormones on retinal cells and their action on vasculature, and found that various hormones are involved in the development and progression of retinopathy in diabetic patients. Androgens and androgen inhibitors appear to play both a causal and a protective role, since they increase blood pressure and the levels of adhesion molecules (ICAM and VCAM-1), with worsening of lipid levels. A low level is associated with the metabolic syndrome and may alter lipid, glycemic, and blood pressure values. Dehydroepiandrosterone has a proven protective effect against the damage of elevated glucose levels in pericytes (142). It is believed that the damage due to elevated glucose levels occurs mainly through oxidative mechanisms. The toxicity of glucose and subsequent vascular damage manifest in four ways: activation of the PKC cascade; activation of aldoso-reductase; protein glycosylation; and activation of the hexosamine pathway. According to the so-called unifying therapy, the formation of free oxygen radicals underlies the mechanisms leading to glucose damage (143, 144).

As oxidative stress is implicated in the development and progression of diabetic retinopathy, studies have investigated the role of estrogens, which possess antioxidant properties. Estrogens stimulate the ERβ receptor and protect retinal cells against oxidative stress through their ability to modulate the transcription of antioxidant genes and protect the mitochondria (145). Estrogens can exert a differential action depending on the stage of retinopathy: during the initial stages, the proliferation of endothelial cells induced by estradiol has a beneficial effect and protects the retina by inducing repair processes, whereas during the proliferative stage, this same effect exacerbates the disease (146, 147). Selective estrogen receptor modulators (SERMs), including tamoxifen and raloxifen, act as antagonists or agonists of estrogen depending on the type of receptor to which they bind. In the retina, both drugs strongly antagonize estrogen-induced angiogenesis (147). From the multitude of data collected so far it is clear that the use of sex hormones or their antagonists in the treatment of retinopathy must be personalized based on sex, age, and stage of disease. Stimulation or hormonal modulation may provide a novel therapeutic option.

Other studies have investigated differences in electroretinographic patterns in relation to neuroretinal function in men and women with type 2 diabetes, but not retinopathy to evaluate risk for neurodegenerative disease (148). The analysis showed that neuroretinal dysfunction leading to diabetic retinopathy was far more common among men than women, suggesting a sex-related protective mechanism. This finding was corroborated in a minireview issued by the Berkeley School of Optometry (49) that noted a more frequent incidence of abnormal neuroretinal function in men with type 2 diabetes. It was also observed that advanced proliferative retinopathy in type 1 diabetes more often affects men, that retinopathy is more likely present at diagnosis of diabetes in men, and that it is often more severe than in women.

#### HRT Does Not Influence Diabetic Retinopathy

A possible benefit of HRT for retinopathy in postmenopausal women has been studied (149); however, no connection between changes in retinopathy and the incidence of macular edema and exposure to exogenous estrogen could be established, indicating that, unlike its effects observed in other eye disorders, HRT has no effect on diabetic retinopathy.

### Glaucoma

#### Benefical Effects of Estrogens in Glaucoma

Glaucoma, the second leading cause of blindness in the world (43), is a slow, progressive neurodegenerative disease characterized by gradual loss of RGC (150) and loss of vision (151). A recent review (152) examining possible correlations between glaucoma and estrogens reported that factors influencing the duration of estrogen exposure (e.g., age at menarche, use of oral contraceptives, bilateral ovariectomy, age at menopause) can raise the risk of POAG. A higher risk for POAG was associated with age at menarche over 13 years, as compared to age less than 12 years (153), and a 25% higher risk was noted in women who took oral contraceptives for more than 5 years (154). Bilateral ovariectomy before age 43 increased the risk of developing glaucoma (155), as did spontaneous menopause before age 45 years (156), whereas the risk was significantly lower in women over age 65 who entered menopause after age 54 years (154). Prolonged estrogen exposure reduced the risk of glaucoma or glaucoma-related conditions, as demonstrated by evidence that IOP is lower during pregnancy when estrogen levels are elevated, particularly during the third trimester (157, 158). Evidence for a protective effect of HRT against glaucoma is uneven (79, 159, 160).

Genetic factors may also play a role in increasing the risk for developing glaucoma. SNPs implicated in the estrogen metabolic pathway associated with women, but not men (161), and polymorphisms of the endothelial nitric oxide synthase gene encoding the enzyme regulated by estrogens are correlated with the development of open-angle glaucoma (162). Estrogen exposure may alter the pathogenesis of glaucoma and exert a neuroprotective action. A future area of focus is the use of estrogens in glaucoma treatment or prevention. Numerous studies have reported an association between estrogens and glaucoma; for example, estrogen deficiency was associated with the acceleration of aging of the optic nerve (163). Estrogens have also been implicated in aqueous humor production and drainage via receptors on the ciliary epithelium (10, 159). Changes in estrogen levels appear to influence IOP, which is responsible for optic nerve trophism (164). Epidemiology, clinical, and experimental evidence supports the hypothesis that early reduction of estrogen levels leads to premature aging of the optic nerve and increased susceptibility to glaucoma.

In their study on the role of estrogens in modulating the topography of the optic nerve head, Akar et al. (165) reported that, as observed in diabetic women, hormonal fluctuations during the menstrual cycle affect the central area and the margin of the optic nerve head. Genetic analysis to determine whether there exist associations between certain SNPs of genes involved in estrogen metabolism and the development of POAG (161) showed that SNPs were correlated with global POAG and POAG with elevated, but not with low IOP and no correlation of any type was noted for the men. Among the women, however, the gene that encodes the catechol-O-methyltransferase enzyme was found to be associated with open-angle glaucoma. Summarizing, links between SNPs of the estrogen pathways and the development of glaucoma were noted for certain types of glaucoma and only in women.

## Future Prospects and Opportunities

### A Place for HRT?

In light of the amount of research conducted so far on sex hormones and neuroretinal diseases, the question arises whether and how it can be applied to the identification and development of new drugs or to expanding indications for drugs already on the market. An emblematic case is that of HRT for the treatment of climacteric symptoms.

Despite the wealth of data, the question remains open. A study evaluated the efficacy of HRT with phytoestrogens on eye function, as measured with short-wavelength automated perimetry in postmenopausal women (166). Phytoestrogens are nonsteroids of plant origin with estrogen-like action in modulating vision sensitivity. Consistent with the theory of timing, which states that the benefits of HRT diminish the later the therapy is initiated, a loss of efficacy was noted in relation to the time between initiation of phytoestrogen therapy and onset of menopausal symptoms, with no benefit gained if the therapy was started in women over 60 years of age. No conclusions could be drawn from this study and no recommendations for the use of HRT for eye diseases could be made.

### Implications for Future Study Design

The present review of the literature found evidence for a sex-related difference in the prevalence of certain eye diseases. A plausible explanation for the difference is the differential effect of sex hormones on the development and course of disease, which may be as meaningful as it is complex, though the underlying mechanisms are not yet fully understood.

The link between sex hormones and retinopathies opens new therapeutic horizons. To obtain a better understanding of the interactions between sex hormones and eye diseases, studies should be designed to determine the presence of an association between sex, hormones, and disease, and if such an association exists, the potential therapeutic correlates. To reach these objectives, key areas of focus are: epidemiological studies on the distribution of eye disease in a population, while taking sex and hormonal status into account; experimental studies on the changes in the incidence and/or course of disease in relation to hormone administration or deprivation; and preclinical animal studies comparing differences between the sexes.

## Author Contributions

All the authors have equally contributed to the search of the literature and cooperated to write the article.

## Conflict of Interest Statement

The authors declare that the research was conducted in the absence of any commercial or financial relationships that could be construed as a potential conflict of interest.

## References

[1] Rosner W Hankinson SE Sluss PM Vesper HW Wierman ME Challenges to the measurement of estradiol: an endrocrine society position statement. J Clin Endocrinol Metab (2013) 98(4):1376–87. 10.1210/jc.2012-3780 23463657 PMC3615207

[2] Clarke BL Khosla S Female reproductive system and bone. Arch Biochem Biophys (2010) 503(1):118–28. 10.1016/j.abb.2010.07.006 20637179 PMC2942975

[3] Gillies GE McArthur S Estrogen actions in the brain and the basis for differential action in men and women: a case for a sex-specific medicines. Pharmacol Rev (2010) 62(2):155–98. 10.1124/pr.109.002071 20392807 PMC2879914

[4] Simpson ER. Sources of estrogen and their importance. J Steroid Biochem Mol Biol (2003) 86(3–5):225–30. 10.1016/S0960-0760(03)00360-1 14623515

[5] Azcoitia I Arevalo MA De Nicola AF Garcia-Segura LM. Neuroprotective actions of estradiol revisited. Trends Endocrinol Metab (2011) 22:467–73. 10.1016/j.tem.2011.08.002 21889354

[6] Melcangi RC Panzica G Garcia-Segura LM. Neuroactive steroids: focus on human brain. Neuroscience (2011) 191:1–5. 10.1016/j.neuroscience.2011.06.024 21704130

[7] Hutchinson CV Walker JA Davidson C Oestrogen, ocular function and low-level vision. J Endocrinol (2014) 223(2):R9–18. 10.1530/JOE-14-0349 25143633

[8] Wickham LA Gao J Toda I Rocha EM Ono M Sullivan DA. Identification of androgen, estrogen and progesterone receptor mRNAs in the eye. Acta Ophthalmol Scand (2000) 78(2):146–53. 10.1034/j.1600-0420.2000.078002146.x 10794246

[9] Gupta PD Johar K Nagpal K Vasavada AR. Sex hormone receptors in the human eye. Surv Ophthalmol (2005) 50(3):274–84. 10.1016/j.survophthal.2005.02.005 15850816

[10] Ogueta SB Schwartz SD Yamashita CK Farber DB. Estrogen receptor in the human eye: influence of gender and age on gene expression. Invest Ophthalmol Vis Sci (1999) 40(9):1906–11. 10440242

[11] Guarnieri P Guarnieri R Cascio C Pavasant P Piccoli F Papadopoulos V Neurosteoidogenesis in rat retina. J Neurochem (1994) 63(1):86–96. 10.1046/j.1471-4159.1994.63010086.x 7911514

[12] Cascio C Russo D Drago G Galizzi G Passantino R Guarnieri R 17b-estradiol synthesis in the adult male rat in retina. Exp Eye Res (2007) 85(1):166–72. 10.1016/j.exer.2007.02.008 17466975

[13] Ishikawa M Yoshitomi T Zorumski CF Izumi Y. Neurosteroids are endogenous neuroprotectants in an ex vivo glaucoma model. Invest Ophthalmol Vis Sci (2014) 55(12):8531–41. 10.1167/iovs.14-15624 25406290 PMC4280088

[14] Cascio C Deidda I Russo D Guarnieri P. The estrogenic retina: the potential contribution to healthy aging and age-related neurodegenerative diseases of the retina. Steroids (2015) 103:31–41. 10.1016/j.steroids.2015.08.002 26265586

[15] Fliesler SJ Bretillon L. The ins and outs of cholesterol in the vertebrate retina. J Lipid Res (2010) 51(12):3399–413. 10.1194/jlr.R010538 20861164 PMC2975712

[16] Mellon SH Griffin LD Compagnone NA. Biosynthesis and action of neurosteroids. Brain Res Brain Res Rev (2001) 37(1–3):3–12. 10.1016/S0165-0173(01)00109-6 11744070

[17] Chaychi S Polosa A Lachapelle P Difference in retinal structure and function between aging male and female Sprague-Dawley rats are strongly influenced by the estrus cycle. PLoS One (2015) 10(8):e0136056 10.1371/journal.pone.0136056 26317201 PMC4552560

[18] Ozawa GY Bearse MA Harrison WW Bronson-Castain K Schneck ME Barez S Differences in neuroretinal function between adult males and females. Optom Vis Sci (2014) 91(6):602–7. 10.1097/OPX.0000000000000255 24748031 PMC4104186

[19] Zetterberg M Age-related eye disease and gender. Maturitas (2016) 83:19–26. 10.1016/j.maturitas.2015.10.005 26508081

[20] Kahn HA Leibowitz HM Ganley JP Kini MM Colton T Nickerson RS The Framingham eye study. I. Outline and major prevalence findings. Am J Epidemiol (1977) 106(1):17–32. 10.1093/oxfordjournals.aje.a112428 879158

[21] Klein BE Klein R Linton KL. Prevalence of age-related lens opacities in a population. The Beaver Dam Eye study. Ophthalmology (1992) 99(4):546–52. 10.1016/S0161-6420(92)31934-7 1584573

[22] Lundstrom M Stenevi U Thorburn W Gender and cataract surgery in Sweden 1992–1997. A retrospective observational study based on the Swedish National Cataract Register. Acta Ophthalmol Scand (1999) 77(2):204–8. 10.1034/j.1600-0420.1999.770218.x 10321540

[23] Lundstorm M Stenevi U Thorburn W The Swedish National Cataract Register: a nine-year review. Acta Ophthalmol Scand (2002) 80(3):248–57. 10.1034/j.1600-0420.2002.800304.x 12059861

[24] Freeman EE Munoz B Schein OD West SK Hormone replacement. Arch Ophthalmol (2001) 119(11):1687–92. 10.1001/archopht.119.11.1687 11709021

[25] Klein BE Lens opacities in women in Beaver Dam, Wisconsin: is there evidence of an effect of sex hormones. Trans Am Ophthalmol Soc (1993) 91:517–44. 8140704 PMC1298481

[26] Klein BE Klein R Ritter LL. Is there evidence of an estrogen effect on age-related lens opacities? The Beaver Dam Eye study. Arch Ophthalmol (1994) 112(1):85–91. 10.1001/archopht.1994.01090130095025 8285900

[27] Younan C Mitchell P Cummin RG Panchapakesan J Rochtchina E Hales AM. Hormone replacement therapy, reproductive factors, and the incidence of cataract and cataract surgery: the Blue Mountains Eye Study. Am J Epidemiol (2002) 155(11):997–1006. 10.1093/aje/155.11.997 12034578

[28] Beebe DC Holekamp NM Shui YB. Oxidative damage and the prevention of age-related cataracts. Ophthalmic Res (2010) 44(3):155–65. 10.1159/000316481 20829639 PMC2952186

[29] Zettemberg M Celojevic D Gender and cataract – the role of estrogen. Curr Eye Res (2015) 40(2):176–90. 10.3109/02713683.2014.898774 24987869

[30] Buch H Nielsen NV Vinding T Jensen GB Prause JU la Cour M 14-years incidence, progression and visual morbidity of age-related maculopathy: the Copenhagen City Eye study. Ophthalmology (2005) 112(5):787–98. 10.1016/j.ophtha.2004.11.040 15878058

[31] Laitinen A Laatikainen L Harkanen T Koskinen S Reunanen A Aromaa A. Prevalence of major eye diseases and causes of visual impairment in the adult Finnish population: a nationwide population-based survey. Acta Ophthalmol (2010) 88(4):463–71. 10.1111/j.1755-3768.2009.01566.x 19878108

[32] Chakravarthy U Wong TY Fletcher A Piault E Evans C Zlateva G Clinical risk factors for age-related macular degeneration: a systematic review and meta-analysis. BMC Ophthalmol (2010) 10:31. 10.1186/1471-2415-10-31 21144031 PMC3009619

[33] Age-Related Eye Disease Study Research Group. Risk factors associated with age-related macular degeneration. A case control study in the age-related eye disease study: age-related eye disease study report number 3. Ophthalmology (2000) 107(12):2224–32. 10.1016/S0161-6420(00)00409-7 11097601 PMC1470467

[34] Rudnicka AR Jarrar Z Wormald R Cook DG Fletcher A Owen CG Age and gender variations in age-related macular degeneration prevalence in populations of European ancestry: a meta-analysis. Ophthalmology (2012) 119(3):571–80. 10.1016/j.ophtha.2011.09.027 22176800

[35] Smith W Mitchell P Wang JJ. Gender, oestrogen, hormone replacement and age-related macular degeneration: results from the Blue Mountains Eye study. Aust N Z J Ophthalmol (1997) 25(Suppl 1):S13–5. 10.1111/j.1442-9071.1997.tb01745.x 9267614

[36] Snow KK Cote J Yang W Davis NJ Seddon JM. Association between reproductive and hormonal factors and age-related maculopathy in postmenopausal women. Am J Ophthalmol (2002) 134(6):842–8. 10.1016/S0002-9394(02)01755-5 12470752

[37] Blasiak J Petrovski G Vereb Z Facsko A Kaarniranta K Oxidative stress, hypoxia and autophagy in the neovascular process of age macular degeneration. Biomed Res Int (2014) 2014:768026 10.1155/2014/768026 24707498 PMC3950832

[38] Kaarniranta K Machalinska A Vereb Z Salminen A Petrovski G Kauppinen A. Estrogen signalling in the pathogenesis of age-related macular degeneration. Curr Eye Res (2015) 40(2):226–33. 10.3109/02713683.2014.925933 24911983

[39] Quigley HA Broman AT. The number of people with glaucoma worldwide in 2010 and 2020. Br J Ophthalmol (2006) 90(3):262–7. 10.1136/bjo.2005.081224 16488940 PMC1856963

[40] Rudnicka AR Mt-Isa S Owen CG Cook DG Ashby D Variation in primary open-angle glaucoma prevalence by age, gender and race: a Bayesian meta-analysis. Invest Ophthalmol Vis Sci (2006) 47(10):4254–61. 10.1167/iovs.06-0299 17003413

[41] Erie JC Hodge DO Gray DT. The incidence of primary angle-closure glaucoma in Olmsted County, Minnesota. Arch Ophthalmol (1997) 115(2):177–81. 10.1001/archopht.1997.01100150179005 9046251

[42] Ng WS Ang GS Azuara-Blanco A Primary angle closure glaucoma: a descriptive study in Scottish Caucasian. Clin Exp Ophthalmol (2008) 36(9):847–51. 10.1111/j.1442-9071.2008.01904.x 19278480

[43] Tham YC Li X Wong TY Quigley HA Aung T Cheng CY. Global prevalence of glaucoma and projections of glaucoma burden through 2040: a systematic review and meta-analysis. Ophthalmology (2014) 121(11):2081–90. 10.1016/j.ophtha.2014.05.013 24974815

[44] Wandell PE Carlsson AC. Time trends and gender differences in incidence and prevalence of type 1 diabetes in Sweden. Curr Diabetes Rev (2013) 9(4):342–9. 10.2174/15733998113099990064 23721159

[45] Chaturvedi N Sjoelie AK Porta M Aldington SJ Fuller JH Songini M Markers of insulin resistance are strong risk factors for retinopathy incidence in type 1 diabetes. Diabetes Care (2001) 24(2):284–9. 10.2337/diacare.24.2.284 11213880

[46] Klein R Knudtson MD Lee KE Gangnon R Klein BE The Wisconsin epidemiologic study of diabetic retinopathy XXIII: the twenty-five-years incidence of macular edema in persons with type 1 diabetes. Ophthalmology (2009) 116(3):497–503. 10.1016/j.ophtha.2008.10.016 19167079 PMC2693093

[47] Kostev K Rathmann W. Diabetic retinopathy at diagnosis of type 2 diabetes in the UK: a database analysis. Diabetologia (2013) 56(1):109–11. 10.1007/s00125-012-2742-7 23052061

[48] Looker HC Nyangoma SO Cromie D Olson JA Leese GP Black J Diabetic retinopathy at diagnosis of type 2 diabetes in Scotland. Diabetologia (2012) 55(9):2335–42. 10.1007/s00125-012-2596-z 22688348 PMC3411303

[49] Ozawa GY Bearse MA Jr Adams AJ. Male-female differences in diabetic retinopathy? Curr Eye Res (2015) 40(2):234–46. 10.3109/02713683.2014.958500 25545999

[50] Nirmalan PK Katz J Robin AL Ramakrishnan R Krishnadas R Thulasiraj RD Female reproductive factors and eye disease in a rural south Indian population: the Aravind comprehensive eye survey. Invest Ophthalmol Vis Sci (2004) 45(12):4273–6. 10.1167/iovs.04-0285 15557432

[51] Nixon E Simpkins JW Neuroprotective effects of nonfeminizing estrogen in retinal photoreceptor neurons. Invest Ophthalmol Vis Sci (2012) 53(8):4739–47. 10.1167/iovs.12-9517 22700711 PMC4625827

[52] Murphy TH Miyamoto M Sastre A Schnaar RL Coyle JT. Glutamate toxicity in a neuronal cell line involves inhibition of cystine transport leading to oxidative stress. Neuron (1989) 2(6):1547–58. 10.1016/0896-6273(89)90043-3 2576375

[53] Bailey TA Kanuga N Romero IA Greenwood J Luthert PJ Cheetham ME. Oxidative stress affects the junctional integrity of retinal pigment epithelial cells. Invest Ophthalmol Vis Sci (2004) 45(2):675–84. 10.1167/iovs.03-0351 14744914

[54] Usui S Oveson BC Lee SY Jo YJ Yoshida T Miki A NADPH oxidase plays a central role in cone cell death in retinitis pigmentosa. J Neurochem (2009) 110(3):1028–37. 10.1111/j.1471-4159.2009.06195.x 19493169 PMC2833098

[55] Mo MS Li HB Wang BY Wang SL Zhu ZL Yu XR PI3K/Akt and NF-kB activation following intravitreal administration of 17b-estradiol: neuroprotection of the rat retina from light-induced apoptosis. Neuroscience (2013) 228:1–12. 10.1016/j.neuroscience.2012.10.002 23069760

[56] Kaja S Yang SH Wei J Fujitani K Liu R Brun-Zinkernagel A Estrogen protects the inner retina from apoptosis and ischemia-induced loss of Vesl-1L/Homer 1c immunoreactive synaptic connections. Invest Ophthalmol Vis Sci (2003) 44(7):3155–62. 10.1167/iovs.02-1204 12824266

[57] Moosmann B Behl C The antioxidant neuroprotective effects of estrogen and phenolic compounds are independent from their estrogenic proprieties. Proc Natl Acad Sci U S A (1999) 96(16):8867–72. 10.1073/pnas.96.16.8867 10430862 PMC17699

[58] Nakazawa T Takakashia H Shimurac M. Estrogen has a neuroprotective effect on axotomized RGCs through ERK signal transduction pathway. Brain Res (2006) 1093(1):141–9. 10.1016/j.brainres.2006.03.084 16696958

[59] Lai P Li T Yang J Xie C Zhu X Xie H Upregulation of stromal cell-derived factor1 (SDF-1) expression in microvasculature endothelial cells in retina ischemia-reperfusion injury. Graefes Arch Clin Exp Ophthalmol (2008) 246(12):1707–13. 10.1371/journal.pone.0114564 18709383

[60] Wang Y Li X Wang J Shi H Bi W Hou W 17b-estradiol mediates upregulation of stromal cell-derived factor-1 in the retina through activation of estrogen receptor in an ischemia-reperfusion injury model. Graefes Arch Clin Exp Ophthalmol (2015) 253(1):17–23. 10.1007/s00417-014-2657-8 24824367

[61] Kaldi I Berta A Progesterone administration fails to protect albino make rats against photostress-induced retinal degeneration. Eur J Ophthalmol (2004) 14(4):306–14. 10.1177/112067210401400405 15309975

[62] Na KS Jee DH Han K Park YG Kim MS Kim EC. The ocular benefits of estrogen replacement therapy: a population-based study in postmenopausal Korean women. PLoS One (2014) 9(9):e106473. 10.1371/journal.pone.0106473 25210892 PMC4161336

[63] Schmidl D Schmetterer L Garhofer G Popa-Cherecheanu A. Gender differences in ocular blood flow. Curr Eye Res (2015) 40(2):201–12. 10.3109/02713683.2014.906625 24892919 PMC4364251

[64] Burgansky-Eliash Z Barash H Nelson D Grinvald A Sorkin A Loewenstein A Retinal blood flow velocity in patient with age-related macular degeneration. Curr Eye Res (2014) 39(3):304–11. 10.3109/02713683.2013.840384 24147793

[65] Grunwald JE Metelitsina TI Dupont JC Ying GS Maguire MG. Reduced foveolar choroidal blood flow in eyes with increasing AMD severity. Invest Ophthalmol Vis Sci (2005) 46(3):1033–8. 10.1167/iovs.04-1050 15728562

[66] Friedman E Krupsky S Lane AM Oak SS Friedman ES Egan K Ocular blood flow velocity in age-related macular degeneration. Ophthalmology (1995) 102(4):640–6. 10.1016/S0161-6420(95)30974-8 7724181

[67] Fuchsjager-Mayrl G Wally B Georgopoulos M Rainer G Kircher K Buehl W Ocular blood flow and systemic blood pressure in patients with primary open-angle glaucoma and ocular hypertension. Invest Ophthalmol Vis Sci (2004) 45(3):834–9. 10.1167/iovs.03-0461 14985298

[68] Meng N Zhang P Huang H Ma J Zhang Y Li H Color Doppler imaging analysis of retrobulbar blood flow velocities in primary open-angle glaucomatous eyes: a meta-analysis. PLoS One (2013) 8(5):e62723. 10.1371/journal.pone.0062723 23675419 PMC3652862

[69] Pemp B Polska E Garhofer G Baverle-Eder M Kautzky-Willer A Schmetterer L. Retinal blood flow in type 1 diabetic patients with no or mild diabetic retinopathy during euglycemic clamp. Diabetes Care (2010) 33(9):2038–42. 10.2337/dc10-0502 20585003 PMC2928359

[70] Patel V Rassam S Newsom R Wiek J Kohner E. Retinal blood flow in diabetic retinopathy. BMJ (1992) 305(6855):678–83. 10.1136/bmj.305.6855.678 1393111 PMC1882919

[71] Gracner T. Ocular blood flow velocity determined by color Doppler imaging in diabetic retinopathy. Ophthalmologica (2004) 218(4):237–42. 10.1159/000078613 15258411

[72] Toker E Yenice O Akpinar I Aribal E Kazokoglu H. The influence of sex hormones on ocular blood flow in women. Acta Ophthalmol Scand (2003) 81(6):617–24. 10.1111/j.1395-3907.2003.00160.x 14641265

[73] Faria AF de Souza MA Geber S. Vascular resistance of central retinal artery is reduced in postmenopausal women after use of estrogen. Menopause (2011) 18(8):869–72. 10.1097/gme.0b013e31820cc60c 21471823

[74] Dechenes MC Descovic D Moreau M Granger L Kuchel GA Mikkola TS Postmenopausal hormone therapy increases retinal blood flow and protects the retinal nerve fiber layer. Invest Ophthalmol Vis Sci (2010) 51(5):2587–600. 10.1167/iovs.09-3710 20019375

[75] Souza AM Souza BM Geber S. Progesterone increases resistance of ophthalmic and central retinal arteries in climacteric women. Climacteric (2013) 16(2):284–7. 10.3109/13697137.2012.720620 23046107

[76] Viana LC Faria M Petternsen H Sampaio M Geber S. Menstrual phase-related differences in the pulsatility index on the central retinal artery suggest an oestrogen vasodilatation effect that antagonizes with progesterone. Arch Gynecol Obstet (2011) 283(3):569–73. 10.1007/s00404-010-1403-7 20213131

[77] Harris-Yitzhak M Harris A Ben-Refael Z Zarfati D Garzozi HJ Martin BJ. Estrogen-replacement therapy: effects on retrobulbar hemodynamics. Am J Ophthalmol (2000) 129(5):623–8. 10.1016/S0002-9394(99)00468-7 10844054

[78] Jones TH. Testosterone deficiency: a risk factor for cardiovascular disease? Trends Endocrinol Metab (2010) 21(8):496–503. 10.1016/j.tem.2010.03.002 20381374

[79] Toker E Yenice O Temel A. Influence of serum levels of sex hormones on intraocular pressure in menopausal women. J Glaucoma (2003) 12(5):436–40. 10.1097/00061198-200310000-00007 14520153

[80] Malan NT Smith W von Kanel R Hamer M Schutte AE Malan L. Low serum testosterone and increased diastolic ocular perfusion pressure: a risk for retinal microvasculature. Vasa (2015) 44(6):435–43. 10.1024/0301-1526/a000466 26515220

[81] Kaarniranta K Sinha D Blasiak J Kauppinen A Vereb Z Salminen A Autophagy and heterophagy dysregulation leads to retinal pigment epithelium dysfunction and development of age-related macular degeneration. Autophagy (2013) 9(7):973–84. 10.4161/auto.24546 23590900 PMC3722332

[82] Eisner A Stoumbos VD Klein ML Fleming SA. Relations between fundus appearance and function. Eyes whose fellow eye has exudative age-related macular degeneration. Invest Ophthalmol Vis Sci (1991) 32(1):8–20. 1987108

[83] Eisner A Klein ML Zilis JD Watkins MD Visual function and the subsequence development of exudative age-related macular degeneration. Invest Ophthalmol Vis Sci (1992) 33(11):3091–102. 1399412

[84] Owsley C McGwin G Jr Jackson GR Kallies K Clark M. Cone- and rod-mediated dark adaptation impairment in age-related maculopathy. Ophthalmology (2007) 114(9):1728–35. 10.1016/j.ophtha.2006.12.023 17822978

[85] Dimitrov PN Robman LD Varsamidis M Aung KZ Makeyeva G Busija L Relationship between clinical macular changes and retinal function in age-related macular degeneration. Invest Ophthalmol Vis Sci (2012) 53(9):5213–20. 10.1167/iovs.11-8958 22714893

[86] Bhutto I Lutty G. Understanding age-related macular degeneration (AMD): relationships between the photoreceptor/retinal pigment epithelium/Bruch’s membrane/choriocapillaris complex. Mol Aspects Med (2012) 33(4):295–317. 10.1016/j.mam.2012.04.005 22542780 PMC3392421

[87] Evans JR. Risk factors for age-related macular degeneration. Prog Retin Eye Res (2001) 20(2):227–53. 10.1016/S1350-9462(00)00023-9 11173253

[88] Mitchell P Wang JJ Foran S Smith W. Five-year incidence of age-related maculopathy lesions: the Blue Mountains Eye study. Ophthalmology (2002) 109(6):1092–7. 10.1016/S0161-6420(02)01055-2 12045049

[89] Fraser-Bell S Wu J Klein R Azen SP Varma R. Smoking, alcohol intake, estrogen use, and age-related macular degeneration in Latinos: the Los Angeles Latino Eye study. Am J Ophthalmol (2006) 141(1):79–87. 10.1016/j.ajo.2005.08.024 16386980

[90] Haan MN Klein R Klein BE Deng Y Blythe LK Seddon JM Hormone therapy and age-related macular degeneration – the women’s health initiative sight exam study. Arch Ophthalmol (2006) 124(7):988–92. 10.1001/archopht.124.7.988 16832022

[91] Feskanich D Cho E Schaumberg DA Colditz GA Hankinson SE. Menopausal and reproductive factors and risk of age-related macular degeneration. Arch Ophthalmol (2008) 126(4):519–24. 10.1001/archopht.126.4.519 18413522

[92] Velez Edwards DE Gallins P Polk M Ayala-Haedo G Schwartz SG Kovach JL Inverse association of female hormone replacement therapy with age-related macular degeneration and interaction with ARMS2 polymorphisms. Invest Ophthalmol Vis Sci (2010) 51(4):1873–9. 10.1167/iovs.09-4000 19933179 PMC2868389

[93] Freeman EE Munoz B Bressler SB West SK. Hormone replacement therapy, reproductive factors and age-related macular degeneration: the Salisbury Eye Evaluation Project. Ophthalmic Epidemiol (2005) 12(1):37–45. 10.1080/09286580490907779 15848919

[94] Virgerling JR Dielemans I Witteman JC Hofman A Grobbee DE de Jong PT Macular degeneration and early menopause: a case-control study. BMJ (1995) 310(6994):1570–1. 10.1136/bmj.310.6994.1570 7787646 PMC2549930

[95] The Royal College of Ophthalmologists. Age-Related Macular Degeneration Guidelines for Management. (2013).

[96] Defay R Pinchinat S Lumbroso S Sutan C Delcourt C Pola Study Group Sex steroid and age-related macular degeneration in older French women: the POLA study. Ann Epidemiol (2004) 14(3):202–8. 10.1016/S1047-2797(03)00130-3 15036224

[97] Abramov Y Borik S Yahalom C Fatum M Avgil G Brzezinski A The effect of the hormone therapy on the risk for age-related maculopathy in postmenopausal women. Menopause (2004) 11(1):62–8. 10.1097/01.GME.0000074701.19603.11 14716184

[98] Klein BE Klein R Lee KE. Reproductive exposures, incident age-related cataracts, and age-related maculopathy in women: the Beaver Dam Eye study. Am J Ophthalmol (2000) 130(3):322–6. 10.1016/S0002-9394(00)00474-8 11020411

[99] Tomany SC Wang JJ Van Leeuwen R Klein R Mitchell P Virgerling JR Risk factors for incident age-related macular degeneration: pooled findings from 3 continents. Ophthalmology (2004) 111(7):1280–7. 10.1016/j.ophtha.2003.11.010 15234127

[100] Chakravarthy U Augood C Bentham GC De Jong PT Rahu M Seland J Cigarette smoking and age-related macular degeneration in the EUREYE study. Ophthalmology (2007) 114(6):1157–63. 10.1016/j.ophtha.2006.09.022 17337063

[101] Wang JJ Klein R Smith W Klein BE Tomany S Mitchell P Cataract surgery and the 5-years incidence of late-stage age-related maculopathy: pooled findings from the Beaver Dam and Blue Mountains eye studies. Ophthalmology (2003) 110(10):1960–7. 10.1016/S0161-6420(03)00816-9 14522772

[102] Cugati S Mitchell P Rochtchina E Tan AG Smith W Wang JJ Cataract surgery and the 10-years incidence of age-related maculopathy: the Blue Mountains Eye study. Ophthalmology (2006) 113(11):2020–5. 10.1016/j.ophtha.2006.05.047 16935334

[103] Klein R Klein BE Wong TY Tomany SC Cruickshanks KJ. The association of cataract and cataract surgery with the long-term incidence of age-related maculopathy: the Beaver Dam Eye study. Arch Ophthalmol (2002) 120(11):1551–8. 10.1001/archopht.120.11.1551 12427071

[104] Hyman LG Lilienfeld AM Ferris FL III Fine SL. Senile macular degeneration: a case-control study. Am J Epidemiol (1983) 118(2):213–27. 10.1093/oxfordjournals.aje.a113629 6881127

[105] Klaver CC Wolfs RC Assink JJ van Dujin CM Hofman A De Jong PT. Genetic risk of age-related maculopathy. Population-based familial aggregation study. Arch Ophthalmol (1998) 116(12):1646–51. 10.1001/archopht.116.12.1646 9869796

[106] Tan JS Mitchell P Smith W Wang JJ Cardiovascular risk factors and the long-term incidence of the age-related macular degeneration: the Blue Mountains Eye study. Ophthalmology (2007) 114(6):1143–50. 10.1016/j.ophtha.2006.09.033 17275090

[107] Iida T Kishi S Hagimura N Shimizu K. Persistent and bilateral choroidal vascular abnormalities in central serous chorioretinopathy. Retina (1999) 19(6):508–12. 10.1097/00006982-199919060-00005 10606450

[108] Prunte C Flammer J. Choroidal capillary and venous congestion in central serous chorioretinopathy. Am J Ophthalmol (1996) 121(1):26–34. 10.1016/S0002-9394(14)70531-8 8554078

[109] Liew G Quin G Gillies M Fraser-Bell S. Central serous chorioretinopathy: a review of epidemiology and pathophysiology. Clin Exp Ophthalmol (2013) 41(2):201–14. 10.1111/j.1442-9071.2012.02848.x 22788735

[110] Ross A Ross AH Mohamed Q. Review and update of central serous chorioretinopathy. Curr Opin Ophthalmol (2011) 22(3):166–73. 10.1097/ICU.0b013e3283459826 21427570

[111] Grieshaber MC Staub JJ Flammer J The potential role of testosterone in central serous chorioretinopathy. Br J Ophthalmol (2007) 91(1):118–9. 10.1136/bjo.2006.098277 17179128 PMC1857578

[112] Haimovici R Rumelt S Melby J. Endocrine abnormalities in patients with central serous chorioretinopathy. Ophthalmology (2003) 110(4):698–703. 10.1016/S0161-6420(02)01975-9 12689888

[113] Zakir SM Shukla M Simi ZUR Ahmad J Sajid M. Serum cortisol and testosterone levels in idiopathic central serous chorioretinopathy. Indian J Ophthalmol (2009) 57(6):419–22. 10.4103/0301-4738.57143 19861741 PMC2812758

[114] Kapetanios AD Donati G Bouzas E Mastorakos G Pournaras CJ Serous central chorioretinopathy and endogenous hypercortisolemia. Klin Monbl Augenheilkd (1998) 212(5):343–4. 10.1055/s-2008-1034901 9677574

[115] Ahad AM Chua CN Evans NM Central serous chorioretinis associated with testosterone therapy. Eye (Lond) (2006) 20(4):503–5. 10.1038/sj.eye.6701905 15846380

[116] Nudleman E Witmer MT Kiss S Wlliams GA Wolfe JD. Central serous chorioretinopathy in patients receiving exogenous testosterone therapy. Retina (2014) 34(10):2128–32. 10.1097/IAE.0000000000000198 24946102

[117] Forooghian F Meleth AD Cukras C Chew EY Wong WT Meyerle CB. Finasteride for chronic central serous chorioretinopathy. Retina (2011) 31(4):766–71. 10.1097/IAE.0b013e3181f04a35 21273946 PMC3116973

[118] Tufan HA Gencer B Comez AT. Serum cortisol and testosterone levels in chronic central serous chorioretinopathy. Graefes Arch Clin Exp Ophthalmol (2013) 251(3):677–80. 10.1007/s00417-012-2075-8 22718204

[119] La Cour M Friis J. Macular holes: classification, epidemiology, natural history and treatment. Acta Ophthalmol Scand (2002) 80(6):579–87. 10.1034/j.1600-0420.2002.800605.x 12485276

[120] Frangieh GT Green WR Engel HM A histopathologic study of macular cyst and holes. Retina (1981) 1(4):311–36. 10.1097/00006982-198101040-00008 7348853

[121] Morgan CM Scha UH. Idiopathic macular holes. Am J Ophthalmol (1985) 99(4):437–44. 10.1016/0002-9394(85)90011-X 3985081

[122] Gass JD Idiopathic senile macular holes: its early stages and pathogenesis. Arch Ophthalmol (1988) 106(5):629–39. 10.1001/archopht.1988.01060130683026 3358729

[123] Wylegała E Woyna-Orlewicz A Piłat J Teper S Ludyga A Traction maculopathies – pathogenesis and diagnostics. Klin Oczna (2006) 108(10–12):457–63. 17455727

[124] McDonnell PJ Fine SL Hillis AI. Clinical features of idiopathic macular cysts and holes. Am J Ophthalmol (1982) 93(6):777–86. 10.1016/0002-9394(82)90474-3 7091263

[125] James M Feman SS. Macular holes. Albrecht Von Graefes Arch Klin Exp Ophthalmol (1980) 215(1):59–63. 10.1007/BF00413397 6906170

[126] Gray RH Gregor ZJ Marsh M Oestrogen and macular holes: a postal questionnaire. Eye (Lond) (1994) 8(3):368–9. 10.1038/eye.1994.83 7958056

[127] McCannel CA Ensminger JL Diehl NN Hodge DN Population based incidence of macular holes. Ophthalmology (2009) 116(7):1366–9. 10.1016/j.ophtha.2009.01.052 19576500 PMC2867090

[128] Evans JR Schwartz SD McHugh JDA Thamby-Rajah Y Hodgson SA Wormald RPL Systemic risk factors for idiopathic macular holes: a case-control study. Eye (Lond) (1998) 12(2):256–9. 10.1038/eye.1998.60 9683950

[129] Chung SE Kim SW Chung HW Kang SW. Estrogen antagonist and development of macular hole. Korean J Ophthalmol (2010) 24(5):306–9. 10.3341/kjo.2010.24.5.306 21052512 PMC2955275

[130] Bourla DH Sarraf D Schwartz SD. Peripheral retinopathy and maculopathy in high-dose tamoxifen therapy. Am J Ophthalmol (2007) 144(1):126–8. 10.1016/j.ajo.2007.03.023 17601434

[131] Inokuchi N Ikeda T Nakamura K Morishita S Fukumoto M Kida T Vitreous estrogen levels in patients with an idiopathic macular hole. Clin Ophthalmol (2015) 9:549–52. 10.2147/OPTH.S80754 25848205 PMC4376308

[132] Usui S Komeima K Lee SY Jo YJ Ueno S Rogers BS Increased expression of catalase and superoxide dismutase 2 reduce con cell death in retinitis pigmentosa. Mol Ther (2009) 17(5):778–86. 10.1038/mt.2009.47 19293779 PMC2803613

[133] Kennan A Aherne A Humpries P. Light in retinitis pigmentosa. Trends Genet (2005) 21(2):103–10. 10.1016/j.tig.2004.12.001 15661356

[134] Doonan F O’Driscoll C Kenna P Cotter TG. Enhancing survival of photoreceptor cells in vivo using the synthetic progestin norgestrel. J Neurochem (2011) 118(5):915–27. 10.1111/j.1471-4159.2011.07354.x 21689103

[135] Doonan F Cotter TG. Norgestrel may be a potential therapy for retinal degenerations. Expert Opin Investig Drugs (2012) 21(5):579–81. 10.1517/13543784.2012.667400 22375616

[136] Sanchez-Vallejo V Benlloch-Navarro S Lopez-Pedrajas R Romero FJ Miranda M. Neuroprotective actions of progesterone in an in vivo model of retinitis pigmentosa. Pharmacol Res (2015) 99:276–88. 10.1016/j.phrs.2015.06.019 26158501

[137] Singh M Su C. Progesterone and neuroprotection. Horm Behav (2013) 63(2):284–90. 10.1016/j.yhbeh.2012.06.003 22732134 PMC3467329

[138] Sivaprasad S Gupta B Crosby-Nwaobi R Evans J. Prevalence of diabetic retinopathy in various ethnic groups: a worldwide perspective. Surv Ophthalmol (2012) 57(4):347–70. 10.1016/j.survophthal.2012.01.004 22542913

[139] Yau JW Rogers SL Kawasaki R Lamoureux EL Kowalski JW Bek T Global prevalence and major risk factors of diabetic retinopathy. Diabetes Care (2012) 35(3):556–64. 10.2337/dc11-1909 22301125 PMC3322721

[140] Romero P Baget M Mendez I Fernandez J Slavat M Martinez I Diabetic macular edema and its relationship to renal microangiopathy: a sample of type 1 diabetes mellitus patients in a 15-year follow up study. J Diabetes Complications (2007) 21(3):172–80. 10.1016/j.jdiacomp.2006.07.008 17493551

[141] Grisby JG Allen DM Culbert RB Escobedo G Parvathaneni K Betts BS The role of sex hormones in diabetic retinopathy. Diabetic Retinopathy (2012) 17:331–56. 10.5772/30643

[142] Briganrdello E Beltramo E Molinatti PA Aragno M Gatto V Tamagno E Dehydroepiandrosterone protects bovine retinal capillary pericytes against glucose toxicity. J Endocrinol (1998) 158(1):21–6. 10.1677/joe.0.1580021 9713322

[143] Cunha-Vaz J Diabetic retinopathy. Human and experimental studies. Trans Ophthalmol Soc U K (1972) 92:111–24. 4515503

[144] Brownlee M. Biochemistry and molecular cell biology of diabetic complications. Nature (2001) 414(6865):813–20. 10.1038/414813a 11742414

[145] Giddabasappa A Bauler M Yepuru M Chaum E Dalton JT Eswaraka J. 17-β estradiol protects ARPE-19 cells from oxidative stress through estrogen receptor-β. Invest Ophthalmol Vis Sci (2010) 51(10):5278–87. 10.1167/iovs.10-5316 20463317

[146] Espinosa-Heidmann DG Marin-Castano ME Pereira-Simon S Hernandez EP Elliot S Cousins SW. Gender and estrogen supplementation increases severity of experimental choroidal neovascularization. Exp Eye Res (2005) 80(3):413–23. 10.1016/j.exer.2004.10.008 15721623

[147] Grigsby JG Parvathaneni K Almaza MA Botello AM Mondragon AA Allen DM Effects of tamoxifen versus raloxifene on retinal capillary endothelial cell proliferation. J Ocul Pharmacol Ther (2011) 27(3):225–33. 10.1089/jop.2010.0171 21413859 PMC3107976

[148] Ozawa GY Bearse MA Bronson-Castain KW Harrison WW Schneck ME Barez S Neurodegenerative difference in the retinas of male and female patients with type 2 diabetes. Invest Ophthalmol Vis Sci (2012) 53(6):3040–6. 10.1167/iovs.11-8226 22491405 PMC3378087

[149] Klein BEK Klein R Moss SE. Exogenous estrogen exposures and changes in diabetic retinopathy: the Wisconsin epidemiologic study of diabetic retinopathy. Diabetes Care (1999) 22(12):1984–7. 10.2337/diacare.22.12.1984 10587830

[150] Schmidt KG Bergert H Funk RH Neurodegenerative disease of the retina and potential for protection and recovery. Curr Neuropharmacol (2008) 6(2):164–78. 10.2174/157015908784533851 19305795 PMC2647152

[151] Yucel YH Zhang Q Weinreb RN Kaufman PL Gupta N. Effects of retinal ganglion cell loss on magno-, parvo-, koniocellular pathways in the lateral geniculate nucleus and visual cortex in glaucoma. Prog Retin Eye Res (2003) 22(4):465–81. 10.1016/S1350-9462(03)00026-0 12742392

[152] Dewundara SS Wiggs JL Sullivan DA Pasquale LR. Is estrogen a therapeutic target for glaucoma? Semin Ophthalmol (2016) 31(1–2):140–6. 10.3109/08820538.2015.1114845 26959139 PMC4930553

[153] Lee AJ Mitchell P Rochtchina E Healey PR Blue Mountains Eye Study. Female reproductive factors and open angle glaucoma: the Blue Mountains Eye study. Br J Ophthalmol (2003) 87(11):1324–8. 10.1136/bjo.87.11.1324 14609824 PMC1771896

[154] Pasquale LR Kang JH Female reproductive factors and primary open-angle glaucoma in Nurses’ Health study. Eye (Lond) (2011) 25(5):633–41. 10.1038/eye.2011.34 21336255 PMC3093442

[155] Vajaranant S Grossardt BR Maki PM Pasquale LR Sit AJ Shuster LT Risk of glaucoma after early bilateral oophorectomy. Menopause (2014) 21(4):391–8. 10.1097/GME.0b013e31829fd081 24061049 PMC3880394

[156] Hulsman CA Westendorp IC Ramrattan RS Wolfs RC Witteman JC Vingerling JR Is open-angle glaucoma associated with early menopause? The Rotterdam study. Am J Epidemiol (2001) 154(2):138–44. 10.1093/aje/154.2.138 11447046

[157] Qureshi IA Xi XR Wu XD. Intraocular pressure trends in pregnancy and in the third trimester hypertensive patients. Acta Obstet Gynecol Scand (1996) 75(9):816–9. 10.3109/00016349609054709 8931505

[158] Qureshi IA. Intraocular pressure: association with menstrual cycle, pregnancy and menopause in apparently healthy women. Chin J Physiol (1995) 38(4):229–34. 8925675

[159] Altinta SO Caglar Y Yuksel N Demirci A Karabas L The effects of menopause and hormone replacement therapy on quality and quantity of tear, intraocular pressure and blood flow. Ophthalmologica (2004) 218(2):120–9. 10.1159/000076148 15004502

[160] Sator MO Joura EA Frigo P Kurz C Metka M Hommer A Hormone replacement therapy and intraocular pressure. Maturitas (1997) 28(1):55–8. 10.1016/S0378-5122(97)00060-1 9391995

[161] Pasquale LR Loomis SJ Weiner RN Kang JH Yaspan BL Bailey JC Estrogen pathway polymorphisms in relation to primary open angle glaucoma: an analysis accounting for gender from the United States. Mol Vis (2013) 19:1471–81. 23869166 PMC3712669

[162] Furchgott RF Zawaszki JV. The obligatory role of endothelial cells in the relaxation of arterial smooth muscle by acetylcholine. Nature (1980) 288(5789):373–6. 10.1038/288373a0 6253831

[163] Vajaranant TS Pasquae LR. Estrogen deficiency accelerates aging of the optic nerve. Menopause (2012) 19(8):942–7. 10.1097/gme.0b013e3182443137 22415565 PMC3376696

[164] Weinreb RN Khaw PT. Primary open-angle glaucoma. Lancet (2004) 363(9422):1711–20. 10.1016/S0140-6736(04)16257-0 15158634

[165] Akar ME Taskin O Yucel I Akar Y The effect of the menstrual cycle on optic nerve had analysis in healthy women. Acta Ophthalmol Scand (2004) 82(6):741–5. 10.1111/j.1600-0420.2004.00351.x 15606474

[166] Eisner A Demirel S. Variability in short-wavelength automated perimetry among peri- or postmenopausal women: a dependence on phyto-oestrogen consumption? Acta Ophthalmol (2011) 89(3):e217–24. 10.1111/j.1755-3768.2009.01799.x 19958290 PMC2888924

